# Deaths

**Published:** 1862-08

**Authors:** 


*Deaths.*—At Madeira, of Phthisis Pulmonalis, on the 16th day of May, Thomas Wakley, Esq., founder and editor of the *London Lancet*, in the 67th year of his age.

